# Arthroscopic-assisted removal of proximal tibia locking plates

**DOI:** 10.1186/1758-2555-4-18

**Published:** 2012-05-28

**Authors:** Justus Gille, Ralf Oheim, Helge Riepenhof, Christine Voigt, Christian Juergens

**Affiliations:** 1 Department of Trauma and Reconstructive Surgery, University of Schleswig-Holstein, Campus Luebeck, 23538 Luebeck, Germany; 2 Department of Trauma Surgery and Sportsmedicine, BG-Traumahospital Hamburg, 21033, Hamburg, Germany; 3 Department of Trauma and Reconstructive Surgery, Diakoniekrankenhaus Friederikenstift gGmbH, Humboldtstraße 5, 30169, Hannover, Germany

**Keywords:** Tibia, Plate, Arthroscopy, Removal

## Abstract

We present a new technique to remove plates from the proximal tibia arthroscopic-assisted with all advantages of the minimally invasive surgery and the possibility to treat concomitant intraarticular pathologies. The initial results (n = 7) are promising with an increase of the Lysholm score in all cases studied [preop. median 78 (range 32–100), postop. median 89 (range 60–100)]. In conclusion, arthroscopic-assisted hardware removal at the proximal tibia is feasible and may provide patients with all the benefits of minimal-invasive hardware removal. The described technique can be recommended for all surgeons familiar with arthroscopic surgery.

## Introduction

Arthroscopic joint surgery has evolved dramatically and become an integral part of orthopedics during the past few decades [1]. Arthroscopic plate removal has only been described after proximal humerus and distal femoral fractures [2,3]. Locking plate fixation is a well established surgical option in tibial plateau fractures and high tibial osteotomies with predominantly good results [4,5]. Nevertheless, complication rates are still high; primary or secondary screw perforations, secondary displacement and sintering of bone and implant impingement in case of malpositioning, a restricted range of motion and persistent pain are the main complication types which indicate a screw or implant removal. Besides this, plate extraction can be performed at the request of patients without any underlying complications. An open implant removal can be an extensive secondary surgery for the patient. A minimal invasive approach with small skin incisions and limited soft tissue trauma seems to be favorable. Additionally, the arthroscopic-assisted approach provides surgeons with an opportunity to treat concomitant intraarticular pathology. We present a novel technique to remove a locking plate from the proximal tibia arthroscopic-assisted and report our initial results.

## Technical note

**Figure 1:**
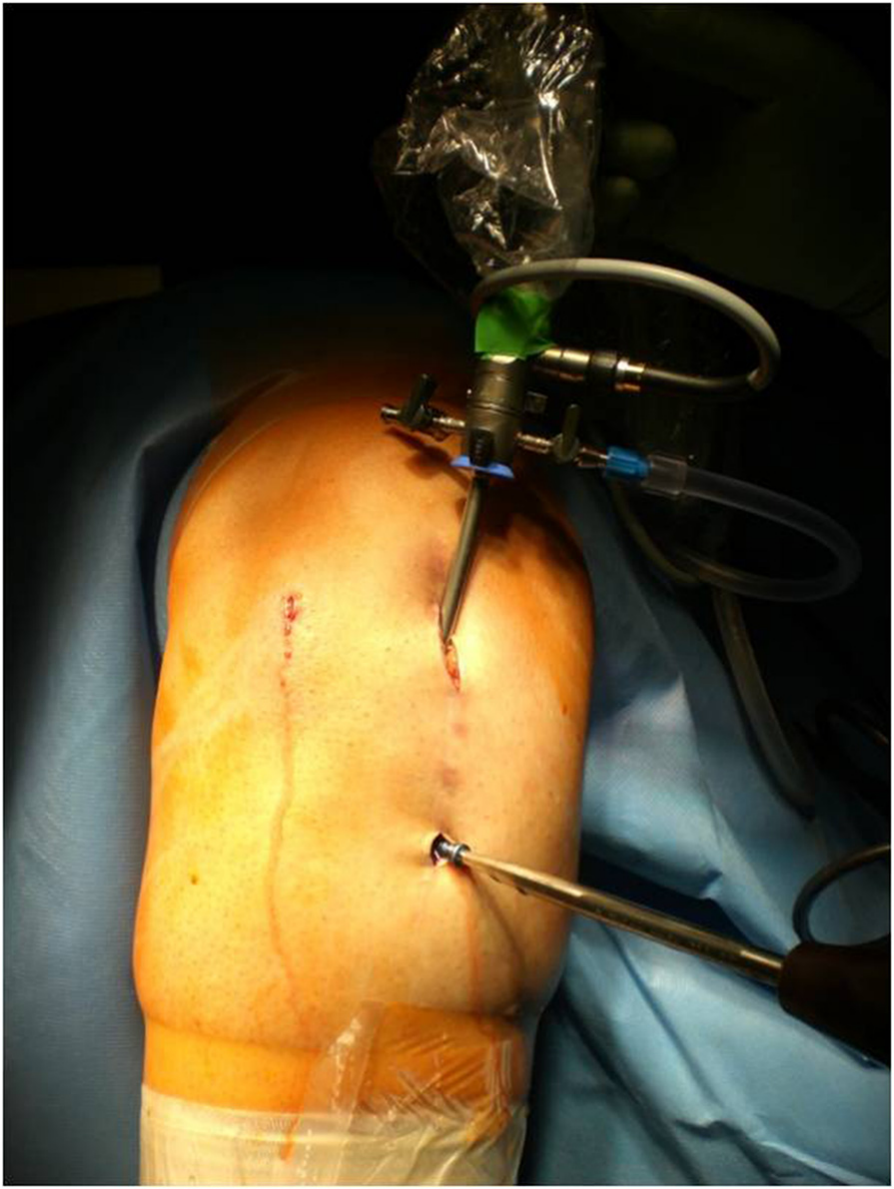
**Right knee, dorsal view.** Surgical access to remove a locking plate arthroscopic-assisted after HTO; the arthroscope is in the standard medial portal.

**Figure 2:**
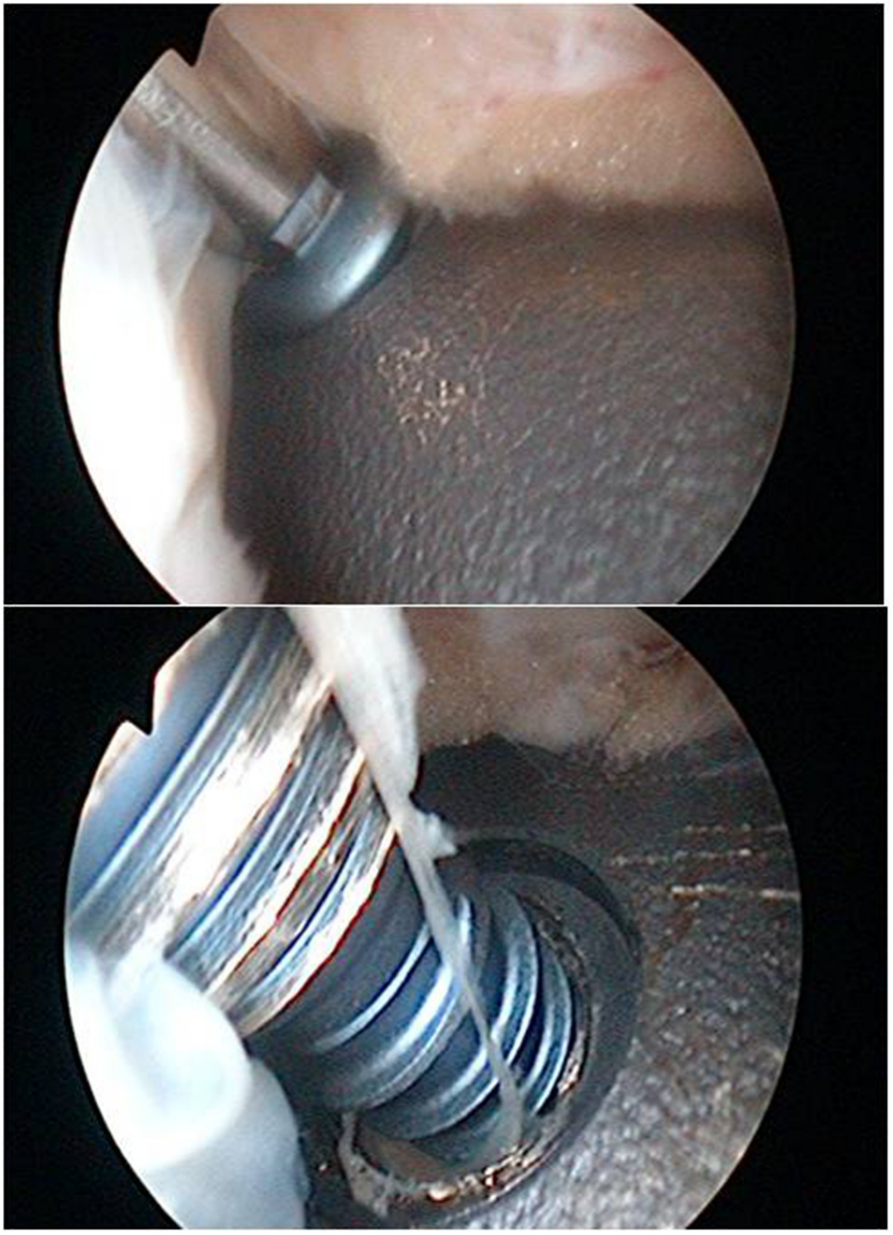
Arthroscopic dorsocaudal view from the anteromedial portal: Taking out a shaft screw with the screwdriver arthroscopic-assisted.

**Figure 3:**
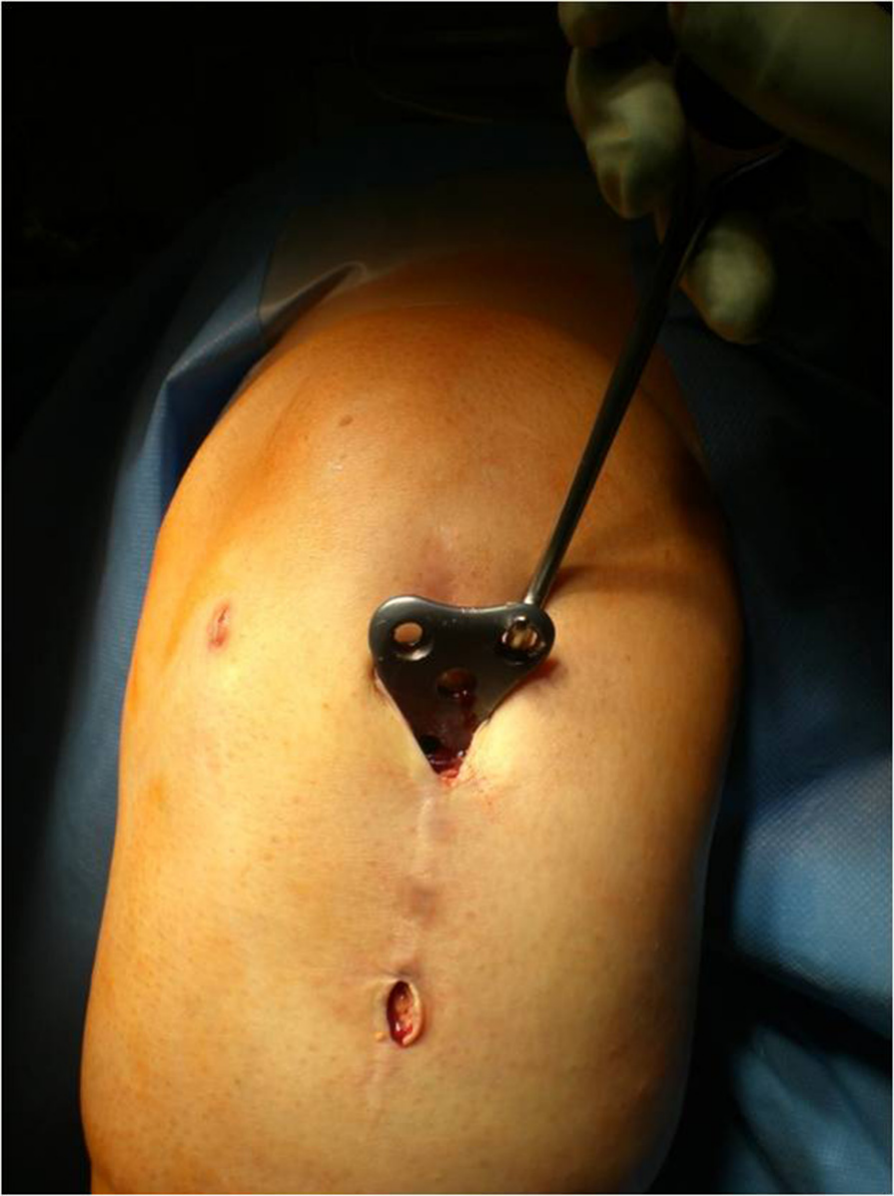
**Right knee, dorsal view.** After taking out the screws, the plate can easily be extracted through the enlarged portal by a hook.

The patient´s leg is fixed in a leg holder. The procedure is done in Esmarch ischaemia. Surgery starts with a diagnostic arthroscopy and concomitant surgery e.g. to address meniscal or cartilage lesions is done. If necessary an arthrolysis adjusted to the limits of motion can be performed, as described before [6]. The portals used are the standard antero-medial and –lateral portal. After arthroscopy of the knee joint is finished, a small raspatorium is used through one of the standard portals to remove soft tissue from the proximal part of the plate. It is our experience that the superior margin of the plate can be easily palpated with the raspatorium. The arthroscope is maintained in this preformed soft tissue cavity and the superior margin of the plate can be defined (Figure 1). The plate is debrided with a shaver, electrodissection or a small rasparatorium close to the plate; an additional incision can be helpful. It is essential to remove soft tissue from screw-head-cones with a small hook to provide good grip of the screwdriver. Then an incision is made so that the proximal screws are located directly under the incision. The screws can be taken out with a screwdriver through this incision (Figure 2). It is very important to pay attention to the correct screw driver direction to avoid losing of the screws in the soft tissue. Depending on the design of the plate, additional small skin incisions between adjacent shaft screws can be necessary. If necessary, ossifications around the margin of the plate can be eliminated by a small chisel over one of the incisions. In the same fashion, the plate can be underrided and lifted. Then one of the proximal skin incisions has to be enlarged and the plate is extracted through this portal with a hook (Figure 3). Finally, the skin incisions are closed and sterile bandage is applied. A postsurgery radiograph is made to document the complete implant removal. Postoperative rehabilitation is early functional without limitations.

## Case series

In a consecutive series of 7 cases hardware from the proximal tibia was removed arthroscopic-assisted, as described above. Inclusion criterion was osseous consolidation after plate fixation in tibia plateau fractures and high tibial osteotomies. Exclusion criteria were patients with adipositas per magna, locking plates, which were implanted several years ago and covered from massive ossifications, or tibial non-unions. The study was performed in compliance with the ethical review board of the University of Luebeck, Germany. In 6 cases initial surgery was performed due to a tibial plateau fracture and in one case a high tibial osteotomy was done. In general, the median age of the patients [3 females, 4 males; mean body mass index 26 (range 20 to 32)] was 43 years (range 24 to 53 years) at the time of the index procedure. Initial surgery was performed at a mean of 1.4 years (range 11 to 26 months) prior to the index procedure. At no point during the procedures the surgeon felt uncomfortable with proceeding arthroscopically and converted to an open procedure. The mean operation time for arthroscopy and arthroscopic-assisted plate removal was 58 minutes (range 37 to 90 minutes). All cases observed did not have any complication associated to the index procedure; none of the patients had any signs of compartment syndrome. Concomitant surgery was done in 6 cases (shaving/microfracture (n = 4); partial meniscal resection (n = 3); arthrolysis (n = 1)). The median follow-up time after the index procedure was 13 month (range 7–18). An increase of the Lysholm score was seen in all cases [preop. median 78 (range 32–100), postop. median 89 (range 60–100)].

### Case report 1

**Figure 4:**
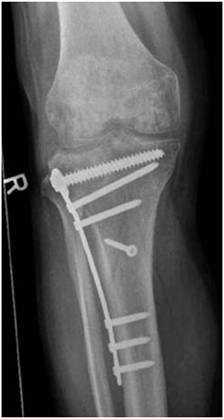
**These radiographs of a 39 year old male (case report 1) were made one year after open reduction and internal fixation.** A good bony consolidation but an incomplete reconstruction of the joint line is visible.

**Figure 5:**
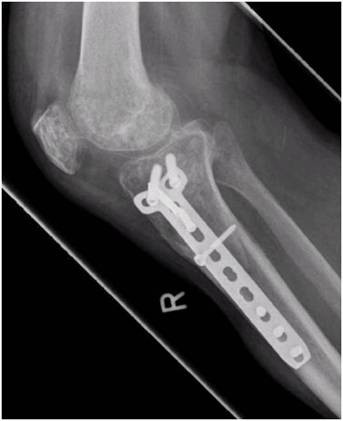
**These radiographs of a 39 year old male (case report 1) were made one year after open reduction and internal fixation.** A good bony consolidation but an incomplete reconstruction of the joint line is visible.

**Figure 6:**
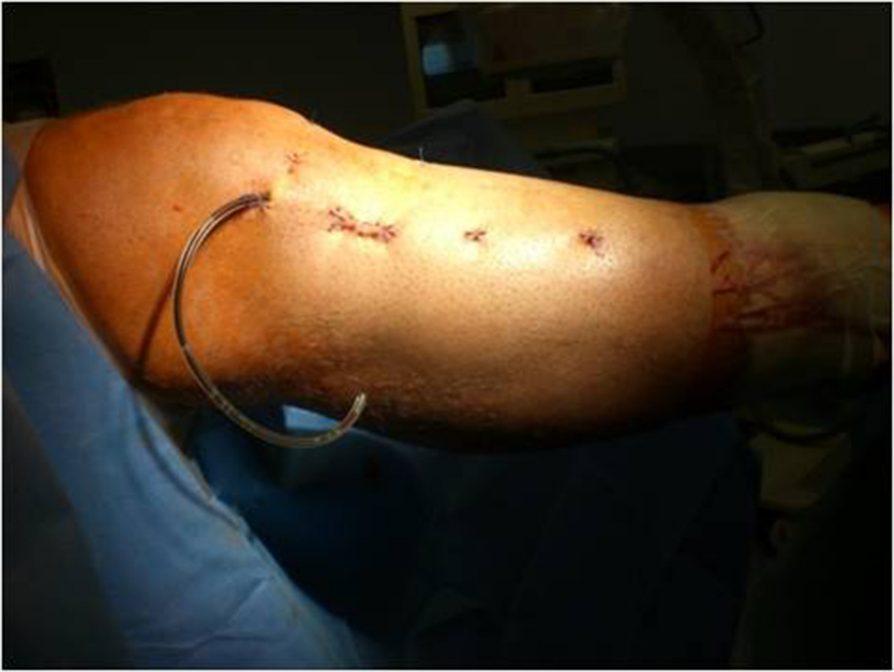
**Right knee and lateral part of the lower leg.** Documentation of the postoperative findings after arthroscopic-assisted plate removal (case report 1).

**Figure 7:**
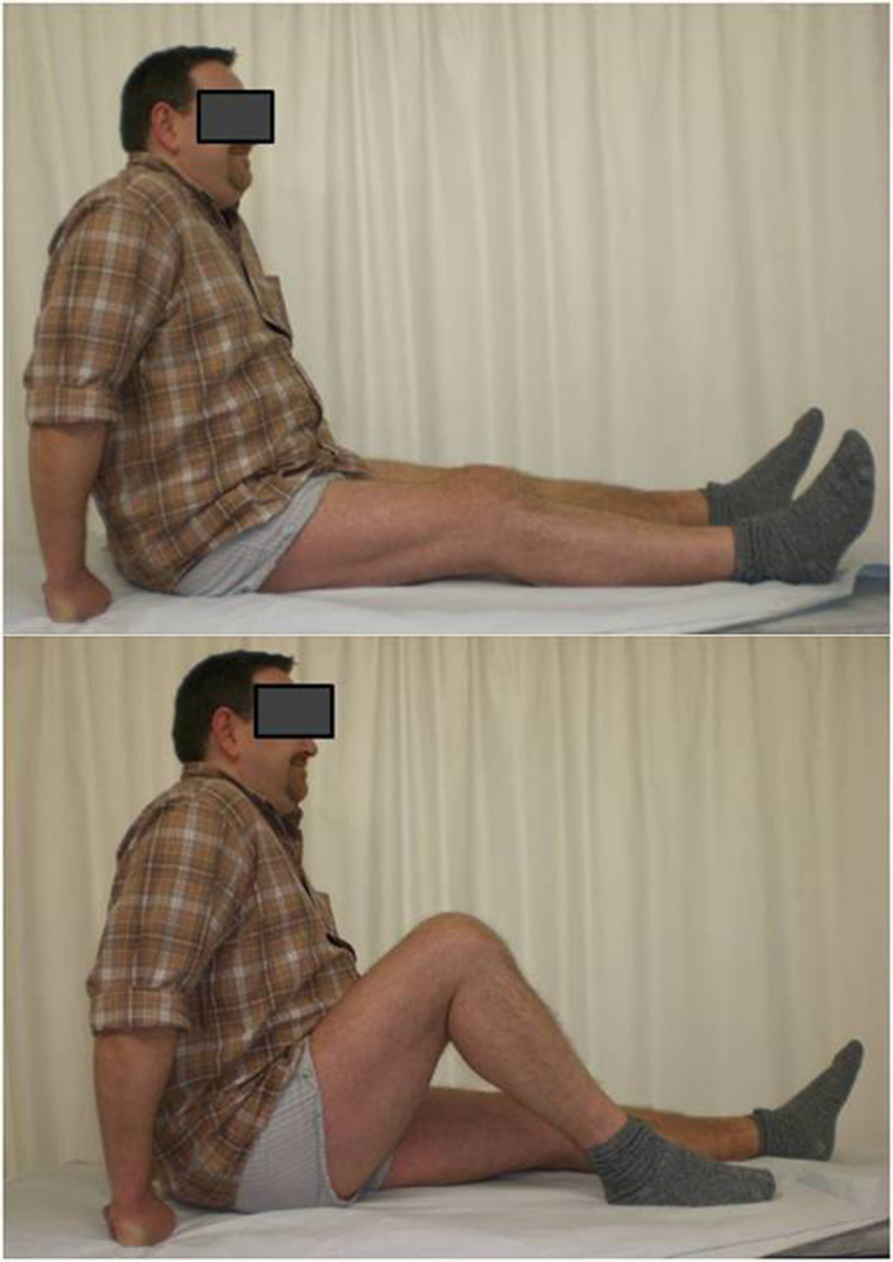
**At the time of clinical follow up 1 year after the index operation the knee shows almost a good range of motion and walking is painfree while fully weightbearing.** The Lysholm score is 84 at follow up.

A Schatzker III tibia plateau fracture (motor vehicle accident) in a 38 y.o. male was addressed with an open reduction and internal fixation with a locked plate in another level I trauma center. On full weight bearing the patient complained of pain with a limited range of motion of the knee. The Lysholm score was calculated with 32 points. One year postoperatively the X-ray showed a good bone consolidation with incomplete reconstruction of the tibial joint line (Figures 4 and 5). An arthroscopy of the knee with an arthrolysis and an arthroscopic-assisted plate removal was done without complications (Figure 6). The patient regained an improved range of movement of the knee [flexion/extension preop.: 90-10-0; 120-5-0 at 1-year follow-up (Figure 7)]. The Lysholm score was calculated with 84 points at one year follow up. To date, the patient returned to work as a mechanic.

### Case report 2

An obese (BMI 27), 24 y.o. female had a high tibial osteotomie (HTO) due to a varus deformity and medial knee chondromalacia. One year postoperatively, the patient elected to undergo implant removal as a result of discomfort over the medial knee joint line and anterior knee pain on extension. At arthroscopy a pathological plica in the knee was resected, before the plate was removed arthroscopic-assisted (Figures 1, 2 and 3). The Lysholm score improved from 62 preoperative to 78 postoperatively at the 1-year-follow-up visit.

## Discussion

The most important finding of the present study is that arthroscopic-assisted removal of hardware following proximal tibial fractures and osteotomies is technically feasible. Addressing the removal of hardware through an arthroscopic-assisted approach enables one to use smaller incisions. Compared to open surgery, the described arthroscopic technique offers advantages:

 minimal soft tissue trauma

 minimal blood loss

 complete knee inspection and treatment of concomitant intraarticular injuries.

The limits of this technique are seen in patients with adipositas per magna, locking plates, which were implanted several years ago and covered from massive ossifications, or fused locking screws, which cannot be grasped by a left-hand thread [2]. In this series we did not experience any possible shortcomings of the arthroscopic-assisted approach (e.g. implant breakage due to poor visualization or incorrect screw driver direction, compartment syndrome, overlong operation time).

To the best of our knowledge this is the first report of arthroscopic-assisted hardware removal at the tibia. Arthroscopic plate removal after proximal humeral fractures has been first described in 2010 [7]. A significant increase of the Constant score was detected at the follow-up examination 6 months postoperatively [2]. In up to 85% patients concomitant intraarticular pathologies were treated parallelly [8]. In a randomized trial arthroscopic implant removal offered all the advantages of minimally invasive surgery and first results were comparable to open implant removal [9].

The arthroscopic-assisted approach provides surgeons with an opportunity to treat concomitant intraarticular pathologies. In a former series, 20-47% of tibial plateau fractures were associated with meniscal lesions [10]. These lesions can be addressed by second-look arthroscopy in combination with the index procedure [11]. Cetik et al. report a high incidence of posttraumatic chondral lesions after tibial plateau fractures [11]. Secondary osteoarthrosis rate is 17–28.9% in the longterm series [12]. The limited healing potential of articular cartilage is a well-known problem in orthopedic surgery [13]. A variety of surgical techniques that aim for resurfacing and regenerating of articular cartilage have evolved [14]. It is open to debate if cartilage repair procedures can decrease the rate of secondary osteoarthritis in the knee.

The presented clinical results at the time of follow-up are in accordance with the literature, reporting good to excellent outcome results in the Lysholm score after tibial plateau fractures [15]. Factors that influence the clinical outcome are articular congruity, stability of the joint, protection of the menisci, coronal alignment, patients’ age and the biological and mechanical behavior of articular cartilage [10]. Our data pinpoint to the fact that clinical results after proximal tibia fractures can be optimized by addressing concomitant lesions, as the Lysholm score results further improved after second-look arthroscopy and plate removal.

There are limitations that need to be acknowledged and addressed regarding the present study. One limitation has to do with the extent to which the findings can be generalized beyond the cases studied. The number of cases is too limited for broad generalization. This study is a consecutive case series with a heterogenous patient population. There is no radiographic follow-up and consequently the development of osteoarthritis cannot be monitored. However, these limitations can be seen as fruitful avenues for future research under the same theme. A randomized trial is on its way and will elucidate the possible advantages of arthroscopic-assisted plate removal in comparison with open surgery at the proximal tibia.

## Conclusion

We conclude that arthroscopic-assisted plate removal at the proximal tibia is feasible and may offer advantages of a minimally invasive surgical procedure to the patient. The described technique can be recommended for all surgeons familiar with arthroscopic surgery.

## Competing interests

The authors declare that they have no competing interests.

## Authors’ contributions

1. Conception and design of the study: JG, CJ 2, Analysis and interpretation of data: RO, HR, CV, JG 3, Collection and assembly of data: RO, HR, CV, JG 4, Drafting of the article: JG, CJ. All authors read and approved the final manuscript.

## References

[B1] Atesok K Doral MN Whipple T Mann G Mei-Dan O Atay OA Beer Y Lowe J Soudry M Schemitsch EH Arthroscopy-assisted fracture fixation Knee Surg Sports Traumatol Arthrosc 2011 19 320 329 10.1007/s00167-010-1298-7 21082166

[B2] Voigt C Geisler A Lill H Arthroscopic locking plate removal after proximal humeral fractures Arch Orthop Trauma Surg 2010 130 391 395 10.1007/s00402-009-0882-0 19387668

[B3] Kim YM Rhee KJ Lee JK Hwang DS Yang JY Kim KC Kim SJ Arthroscopy-assisted minimally invasive removal of a plate in the distal femur Arthroscopy 2006 22 1362 e1361 e1364 10.1016/j.arthro.2006.05.029 17157740

[B4] Stannard JP Finkemeier CG Lee J Kregor PJ Utilization of the less-invasive stabilization system internal fixator for open fractures of the proximal tibia: a multi-center evaluation Indian J Orthop 2008 42 426 430 10.4103/0019-5413.43390 19753230 PMC2740351

[B5] Iorio R Pagnottelli M Vadala A Giannetti S Di Sette P Papandrea P Conteduca F Ferretti A Open-wedge high tibial osteotomy: comparison between manual and computer-assisted techniques Knee Surg Sports Traumatol Arthrosc 2011 (epub ahead of print) 10.1007/s00167-011-1785-5 22113220

[B6] Said S Christainsen SE Faunoe P Lund B Lind M Outcome of surgical treatment of arthrofibrosis following ligament reconstruction Knee Surg Sports Traumatol Arthrosc 2011 19 1704 1708 10.1007/s00167-011-1472-6 21432620

[B7] Dines JS Hettrich CM Kelly BT Savoie FH Lorich DG Arthroscopic removal of proximal humerus locking plates Knee Surg Sports Traumatol Arthrosc 2010 18 409 411 10.1007/s00167-009-0991-x 19946666

[B8] Lill H Katthagen C Voigt C Technique and value of arthroscopic implant removal in the shoulder Orthopade 2011 40 79 84 10.1007/s00132-010-1683-4 21161167

[B9] Katthagen JC Jensen G Hennecke D Lill H Voigt C Arthroscopic implant removal after fixed-angle plate osteosynthesis of the proximal humerus : Technique and initial results in comparison to open implant removal Unfallchirurg 2012 115 1 47 54 10.1007/s00113-011-1953-6 21604039

[B10] Marsh JL Buckwalter J Gelberman R Dirschl D Olson S Brown T Llinias A Articular fractures: does an anatomic reduction really change the result? J Bone Joint Surg Am 2002 84-A 1259 1271 12107331

[B11] Cetik O Cift H Asik M Second-look arthroscopy after arthroscopy-assisted treatment of tibial plateau fractures Knee Surg Sports Traumatol Arthrosc 2007 15 747 752 10.1007/s00167-006-0276-6 17225173

[B12] Roerdink WH Oskam J Vierhout PA Arthroscopically assisted osteosynthesis of tibial plateau fractures in patients older than 55 years Arthroscopy 2001 17 826 831 11600979 10.1016/s0749-8063(01)90005-2

[B13] Ossendorf C Kaps C Kreuz PC Burmester GR Sittinger M Erggelet C Treatment of posttraumatic and focal osteoarthritic cartilage defects of the knee with autologous polymer-based three-dimensional chondrocyte grafts: 2-year clinical results Arthritis Res Ther 2007 9 R41 10.1186/ar2180 17451597 PMC1906819

[B14] Gille J Schuseil E Wimmer J Gellissen J Schulz AP Behrens P Mid-term results of Autologous Matrix-Induced Chondrogenesis for treatment of focal cartilage defects in the knee Knee Surg Sports Traumatol Arthrosc 2010 18 1456 1464 10.1007/s00167-010-1042-3 20127072

[B15] Partenheimer A Gosling T Muller M Schirmer C Kaab M Matschke S Ryf C Renner N Wiebking U Krettek C Management of bicondylar fractures of the tibial plateau with unilateral fixed-angle plate fixation Unfallchirurg 2007 110 675 683 10.1007/s00113-007-1271-1 17497119

